# Endoplasmic reticulum stress triggers ROS signalling, changes the redox state, and regulates the antioxidant defence of *Arabidopsis thaliana*

**DOI:** 10.1093/jxb/eru034

**Published:** 2014-02-20

**Authors:** Rengin Ozgur, Ismail Turkan, Baris Uzilday, Askim H. Sekmen

**Affiliations:** Department of Biology, Faculty of Science, Ege University, Bornova, 35100, Izmir, Turkey

**Keywords:** Antioxidant defence, endoplasmic reticulum stress, ROS signalling, oxidative stress, salt stress, tunicamycin.

## Abstract

Inefficient chaperone activity in endoplasmic reticulum (ER) causes accumulation of unfolded proteins and is called ER stress, which triggers the unfolded protein response. For proper oxidative protein folding, reactive oxygen species (ROS) such as H_2_O_2_ are produced in the ER. Although the role of ROS during abiotic stresses such as salinity is well documented, the role of ER-related ROS production and its signalling is not yet known. Moreover, how H_2_O_2_ production, redox regulation, and antioxidant defence are affected in salt-treated plants when ER protein-folding machinery is impaired needs to be elucidated. For this aim, changes in NADPH-oxidase-dependent ROS signalling and H_2_O_2_ content at sequential time intervals and after 48h of ER stress, induced by tunicamycin (Tm), salinity, and their combination were determined in *Arabidopsis thaliana*. The main root growth was inhibited by ER stress, while low levels of Tm caused an increase in lateral root density. Salt stress and Tm induced the expression of ER-stress-related genes (*bZIP17*, *bZIP28*, *bZIP60*, *TIN1*, *BiP1*, *BiP3*) and *ERO1*. Tm induced expression of *RBOHD* and *RBOHF*, which led to an early increase in H_2_O_2_ and triggered ROS signalling. This study is the first report that ER stress induces the antioxidant system and the Asada–Halliwell pathway of *A. thaliana* in a similar way to salinity. ER stress caused oxidative damage, as evident by increased H_2_O_2_ accumulation, lipid peroxidation, and protein oxidation. As a result, this study shows that ER stress triggers ROS signalling, changes the redox state, and regulates the antioxidant defence of *A. thaliana*.

## Summary

Endoplasmic reticulum stress, which is induced by tunicamycin, triggers reactive oxygen species signalling via NADPH oxidase activity and also regulates the antioxidant defence system in *Arabidopsis thaliana*.

## Introduction

Secretory and membrane proteins synthesized in rough endoplasmic reticulum (ER) of eukaryotic cells must undergo proper folding and modification, such as carbohydrate addition and disulphide bond formation, in the ER before they are transferred via the Golgi to their final destinations (Lai *et al.*, 2007; Urade, 2007). The ER provides a unique oxidizing environment for protein folding and disulphide bond formation (Schröder and Kaufman, 2005). ER oxidoreductase 1 (ERO1), a glycosylated flavoenzyme tightly associated with the lumenal face of the ER membrane, catalyses the formation of disulphide bonds. It is a significant source of oxidizing equivalents for the ER lumen and thus is responsible for regulating ER oxidation state (Pollard *et al.*, 1998). ERO1 cooperates with a thioredoxine-like protein, protein disulphide isomerase, to transfer disulphides to secretory proteins. ERO1 affects protein oxidation by coupling the oxidizing power of molecular oxygen and its flavin cofactor to form disulphide bonds (Gross *et al.*, 2006; Sevier and Kaiser, 2008) and, during this process, oxidant hydrogen peroxide (H_2_O_2_) is formed. It has been suggested that oxidation of cysteine residues during disulphide bond formation in the ER may significantly contribute to oxidative stress in animals (Harding *et al.*, 2003; Tu and Weissman, 2004).

Under adverse environmental conditions such as an onset of abiotic stress (i.e. heat, drought, salt, and a heavy secretory load), the demand on the cell for protein folding can exceed its folding capacity and lead to the accumulation of unfolded or misfolded proteins (Iwata and Koizumi, 2012; Howell, 2013). Accumulation of unfolded proteins causes ER stress and, in turn, induces the unfolded protein response (UPR), which is a significant adaptive signalling pathway designed to prevent accumulation of misfolded proteins in the ER lumen. Studies also suggest that the UPR minimizes the stress of oxidative protein folding in animals (Malhotra and Kaufman, 2007).

As a part of UPR signalling, enhanced ERO1 activity in the ER due to an increase in demand for protein folding and modification induces glutathione oxidation (GSSG formation) (Cabibbo *et al.*, 2000; Sevier *et al.*, 2007) and also impacts glutathione synthesis in animals (Molteni *et al.*, 2004). It has been suggested that GSSG formation in the ER reflects glutathione-mediated reduction of misoxidized substrate proteins and/or protein disulphide isomerase (Tu *et al.*, 2000; Jessop *et al.*, 2004). Thus, ERO1 activity would both generate reactive oxygen species (ROS) and deplete a scavenger of ROS, reduced glutathione. Increased glutathione synthesis under oxidative stress may allow for ROS inactivation, even in the presence of an increased rate of glutathione oxidation. Accordingly, addition of exogenous glutathione reduces UPR activation and prevents ROS accumulation under conditions that normally lead to enhancement of ROS levels in the ER lumen in animals (Harding *et al.*, 2003; Haynes *et al.*, 2004). These findings in animals indicate that ROS are a signal generated by misfolded proteins in the ER that causes UPR activation and cell death. However, how protein misfolding and oxidative stress impact each other has not yet been explored in plant systems.

Iwata and Koizumi (2005) found that exogenous application of an antibiotic, tunicamycin (Tm), which artificially inhibits protein folding by being an inhibitor of N- glycosylation, increased expression of some UPR genes, such as *bZIP60* (*basic leucine zipper 60*), *BiP1* (*Binding Protein1*), and *BiP3* (*Binding Protein3*) in *Arabidopsis thaliana* (Iwata *et al.*, 2008). The same research group also characterized a specific gene, *TIN1* (Tunicamycin Induced 1), which responds directly to ER stress caused by Tm (Iwata *et al.*, 2010). These genes are part of a set of genes taking part in the UPR that increases protein-folding capacity and some responses that alleviate ER stress. Moreover, not only Tm but also abiotic stresses are likely to have an effect on membrane functions and ER-bound transcription factors which have been shown to mediate abiotic stress responses (Jaspers and Kangasjärvi, 2010). Liu *et al.* (2007) reported a relation between salt and ER stress, but there is no study to show a link between the role of ER-originated ROS and sensing of stress signalling. Hence, it is also tempting to investigate the possible interaction between ROS and UPR induction under abiotic stresses such as salinity.

This study investigated the differences and similarities in H_2_O_2_ production, redox regulation, and antioxidant defence under both ER stress and salt stress in *Arabidopsis* plants. This study also provides data about how H_2_O_2_ production, redox regulation, and antioxidant defence are affected in salt-treated plants when the ER protein-folding machinery is impaired. Under the effects of Tm, salt, and combinations of salt and Tm, changes in NADPH oxidase (NOX)-dependent ROS signalling (NOX activity and *RBOHD* and *RBOHF* expression) and H_2_O_2_ content at sequential time intervals (at 10, 30, 60min, and 6 and 24h) were determined, and the effects of Tm on root growth and indicators of stomatal closure were investigated. The roles of the antioxidant defence system and redox status [superoxide dismutase (SOD), catalase (CAT), ascorbate peroxidase (APX), and glutathione reductase (GR), glutathione (GSH), and oxidized glutathione (GSSG)], under salt and Tm were revealed. Moreover, changes in expression of ER-stress-related genes were also identified.

## Materials and methods

### Plant material, growth conditions, and stress treatments

In this study, *A. thaliana* ecotype Col-0 was used as plant material. Plants were grown in a plant growth chamber using a hydroponic system under controlled conditions (12/12h light/dark cycle, 23/21 °C, relative humidity 60%, and light intensity 200 μmol photon m^–2^ s^–1^) with half-strength Hoagland’s solution. After 3 weeks of growth, plants were treated with 80mM NaCl for salt treatment, 1 μg ml^–1^ Tm for ER stress, or 80mM NaCl with 1 μg ml^–1^ Tm for combined stress. Both NaCl and Tm were added to the Hoagland’s solution. For time-course analysis, plants were harvested at 10, 30, 60min, and 6 and 24h of treatment. For other analysis, plants were harvested 48h of treatment. Harvested plants were frozen in liquid nitrogen and were stored at –80 °C until further analysis.

### Root phenotype analysis

Surface-sterilized (70% ethanol and 4% bleach) seeds were germinated in half-strength MS medium and were transferred to half-strength MS plates containing 80mM NaCl or 0.1, 0.25, or 1 μg ml^–1^ Tm or their combination. Plants were grown vertically for 6 d and roots were scanned. Root pictures were analysed using EZ-Rhizo software (Armengaud *et al.*, 2009) to calculate main root length and lateral root density. At least six different plants were used per treatment group.

### Indicators of plant water status and stomatal closing

#### Leaf osmotic potential

Leaf osmotic potential was measured using a Vapro Vapor pressure Osmometer 5520. The data were collected from six plants per replicate.

#### Leaf relative water content

Whole rosettes (*n*=6) were obtained from each treatment group and their freshweights (FW) were determined. The rosettes were floated on deionized water for 6h under low irradiance and then the turgid tissue was quickly blotted to remove excess water and their turgid weights (TW) were determined. Dry weights (DW) were determined after the leaves were dried in the oven. The relative water content (%) was calculated using the following formula: (FW–DW)/(TW–DW)×100.

#### Leaf water loss

For determination of leaf water loss, leaves were detached from the plants and their weights were measured immediately and after 10 and 20min in the growth chamber. Leaf water loss was calculated as the percentage of the original weight of the leaves. Six replicates were used from different plants per treatment group.

### Quantitative reverse-transcription PCR

RNA was isolated from 0.1g fresh tissue using TRIzol reagent (Invitrogen) according to manufacturer’s directions. Total RNA was treated with DNAse I (Fermentas) to remove residual genomic DNA. Then, reverse-transcription PCR was done (1 μg total RNA for each treatment group) using M-MuLV reverse transcriptase (New England Biolabs) and the cDNA was used as a template for quantitative reverse-transcription-PCR (qRT-PCR) using Maxima SYBR Green qPCR Master Mix (Thermo Scientific) and a iQ5 Real-Time PCR system (Bio-Rad). The amounts of RNA in each reaction were normalized with *A. thaliana Actin8*. The conditions for amplification were as follows: 95 °C for 5min and 40 cycles at 94 °C for 15 s, 58 °C for 15 s, and 72 °C for 30 s. Three independent experiments were performed and data analysis was performed with iQ5 software using Pfaffl’s model (Pfaffl, 2001). Control plants were used as the reference point (set to 1) and relative expression levels were calculated with respect to this reference value. The primers for *Actin8*, *bZIP17*, *bZIP28*, *bZIP60*, *BiP1*, *BiP3*, *TIN1*, *ERO1*, *RBOHD*, and *RBOHF* are given in Supplementary Table S1 (available at *JXB* online). The primers were synthesized by Sentromer DNA Technologies (Istanbul, Turkey).

### Enzyme extraction and assays

Enzyme extraction was performed at 4 °C. Samples (0.1g) were ground to a fine powder in liquid nitrogen and then homogenized in 500 μl 50mM TRIS-HCl (pH 7.8) containing 0.1mM EDTA, 0.1% (w/v) Triton-X100, 1mM PMSF, and 1% PVP (w/v) For APX activity determination, 5mM ascorbate was added into the homogenization buffer. Samples were centrifuged at 14,000 *g* for 10min, and supernatants were used for the determination of protein content and enzyme activities. Total soluble protein contents of the enzyme extracts were determined according to Bradford (1976) using BSA as a standard. All spectrophotometric analyses were conducted on a Shimadzu UV 1700 spectrophotometer.

SOD (EC 1.15.1.1) activity was assayed by its ability to inhibit photochemical reduction of nitroblue tetrazolium (NBT) at 560nm (Beauchamp and Fridovich, 1971). One unit of SOD was defined as the amount of enzyme that inhibited 50% NBT photoreduction. CAT (EC 1.11.1.6) activity was estimated according to the method of Bergmeyer (1970), which measures the initial rate of decomposition of H_2_O_2_ at 240nm. The decrease in the absorption was followed for 1min, and 1 μmol H_2_O_2_ min^−1^ was defined as 1 unit of CAT. APX (EC 1.11.1.11) activity was measured according to Nakano and Asada (1981). The assay depends on the decrease in absorbance at 290nm as ascorbate is oxidized. The concentration of oxidized ascorbate was calculated using an extinction coefficient of 2.8mM^−1^ cm^−1^. One unit of APX was defined as 1 μmol ascorbate oxidized min^−1^. GR (EC 1.6.4.2) activity was measured according to Foyer and Halliwell (1976). NADPH oxidation was followed at 340nm. Activity was calculated using the extinction coefficient of NADPH (6.2mM^−1^ cm^−1^). One unit of GR was defined as 1 μmol GSSG reduced min^−1^. NOX (EC 1.6.3. 1) activity was measured according to Jiang and Zhang (2002). The assay medium contained 50mM TRIS-HCl buffer (pH 7.5), 0.5mM XTT, and 100 μM NADPH, and after the addition of NADPH, XTT reduction was followed at 470nm. The corrections for background reduction were determined in the presence of 50U SOD. Activity was calculated using the extinction coefficient 2.16×10^4^ M^−1^ cm^−1^. One unit of NOX was defined as 1 μmol XTT reduced min^−1^. The specific enzyme activity for all enzymes was expressed as U (mg protein)^−1^.

#### Identification of isoenzymes

Samples containing equal amounts of protein were subjected to native polyacrylamide gel electrophoresis as described by Laemmli (1970). For the separation of SOD isoenzymes, 4.5% stacking and 12.5% separating gels were used. SOD activity was detected as described by Beauchamp and Fridovich (1973). The different types of SOD were differentiated by incubating gels in inhibitors of SOD before staining, for example with 2mM KCN to inhibit Cu/ZnSOD activity and with 3mM H_2_O_2_ to inhibit Cu/ZnSOD and FeSOD activities, as described by Vitória *et al.* (2001); MnSOD activity is resistant to both inhibitors. CAT isoforms were detected according to Woodbury *et al.* (1971). The electrophoretic separation was performed using 7.5% separating gels. The gels were incubated in 0.01% H_2_O_2_ for 5min. After incubation, the gels were washed with distilled water twice and incubated for 5min in staining solution containing 1% FeCl_3_ and 1% K_3_Fe(CN)_6_. GR isoforms were detected using 7.5% separating gels according to Hou *et al.* (2004). GR isoforms were detected by incubating the gels in a solution containing 10mM TRIS-HCl (pH 7.9), 4mM GSSG, 1.5mM NADPH, and 2mM DTNB for 20min. After a brief rinse with 50mM TRIS-HCl buffer (pH 7.9), GR activity was negatively stained by 1.2mM MTT and 1.6mM PMS for 5–10min at room temperature. Gels were photographed with a gel imaging system and then analysed with BioCapt software (Vilber Lourmat, Marne la Vallée, France).

### Parameters related to oxidative stress

#### GSH and GSSG

GSH and GSSG contents were determined according to Queval and Noctor (2007). Extractions were performed at 4 °C. Leaf tissue (0.1g) was ground in liquid nitrogen and extracted with 1ml 0.2M HCl. After this, samples were centrifuged at 16 000 *g* for 10min. Supernatant (0.5ml) was neutralized with approximately 0.4ml of 0.2M NaOH in the presence of 50 μl of 0.2M NaH_2_PO_4_ (pH 5.6). The pH of the neutralized acid extracts was between 5 and 6. Glutathione content was determined using an enzyme cycling assay and following the change in absorbance at 340nm, and GSSG was determined using 2-vinylpyridine derivatization followed by an enzyme cycling assay.

#### H_2_O_2_

H_2_O_2_ was determined according to Cheeseman (2006) using eFOX reagent, which is widely used throughout the literature. This modified ferrous ammonium sulphate/xylenol orange (FOX) assay was used due to its sensitivity, stability, and its adaptability to a large number of samples. In this assay 1% ethanol is added to the reagent, which increases its sensitivity to H_2_O_2_ by 50% (i.e. eFOX). In addition, this assay was also adapted to commercial H_2_O_2_ kits due to its sensitivity for determination of H_2_O_2_ content. Extraction was done using ice-cold acetone containing 25mM H_2_SO_4_. Samples were centrifuged for 5min at 3000 *g* at 4 °C. For 50 μl of supernatant, 950 μl eFOX reagent (250 μM ferrous ammonium sulphate, 100 μM xylenol orange, 100 μM sorbitol, 1% ethanol, v/v) was used. Reaction mixtures were incubated at room temperature for 30min and then absorbance at 550 and 800nm was measured. H_2_O_2_ concentrations were calculated using a standard curve prepared with known concentrations of H_2_O_2_.

#### Lipid peroxidation

The level of lipid peroxidation in samples was determined in terms of thiobarbituric acid reactive substances (TBARS) according to the method of Madhava Rao and Sresty (2000).

#### Protein oxidation

Oxidative damage on proteins was measured by determining the level of protein carbonyl groups in the samples using the DPNH derivatization method (Levine *et al.*, 1994).

### Statistical analysis

The results were expressed as means, and error bars were used to show SEM. Groups were compared by t-test using GraphPad 6 statistics software. Asterisks in graphs indicate significant differences between control and treatment groups (*P*<0.05).

## Results

### ER stress inhibited main root growth; however, low levels of Tm caused an increase in lateral root density

**Fig. 1.**
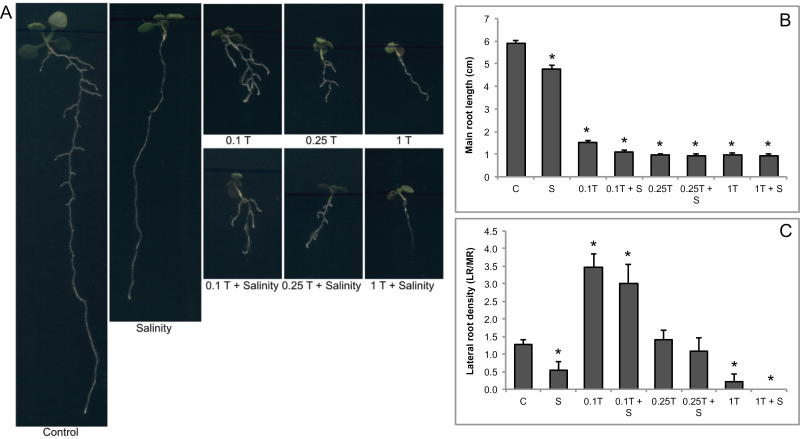
Root phenotype data for controls and plants treated with 80mM NaCl and/or tunicamycin (0.1, 0.25, and 1 μg ml^–1^). (A) Representative images. (B) Main root length. (C) Lateral root density. At least six plants were used for calculations. C, control; S, salinity; T, tunicamycin (this figure is available in colour at *JXB* online).

It is known that stress conditions affect root phenotypes of *Arabidopsis* (Benfey *et al.*, 2010). The ER-stress inducer Tm also inhibits root growth, but there is no information on the interaction between salt stress and ER stress on root phenotypes. To test this, germinated seedlings were transferred to plates containing 80mM NaCl, different Tm concentrations (0.1, 0.25, and 1 μg ml^–1^), and combinations of these treatments (80mM NaCl with 0.1 μg ml^–1^, 80mM NaCl with 0.25 μg ml^–1^, 80mM NaCl with 1 μg ml^–1^; Fig. 1A). Tm treatment greatly decreased main root length, whereas there was only 20% decrease in salt-treated plants (Fig. 1B). To see how Tm changed the root growth response to salinity, the combinations of these stresses were applied to germinated seedlings. A higher decrease in main root growth was detected under the combination of Tm and salt stresses (Fig. 1B).

Salt stress also decreased lateral root density by 59% as compared to control, similarly to its effect on main root length (Fig. 1C). On the other hand, while Tm treatment highly decreased main root length and 1 μg ml^–1^ Tm completely inhibited lateral root formation, 0.1 μg ml^–1^ Tm increased lateral root density by 2.7-fold, as compared to controls (Fig. 1C).

### Tm-induced ER stress decreased leaf water loss

**Fig. 2.**
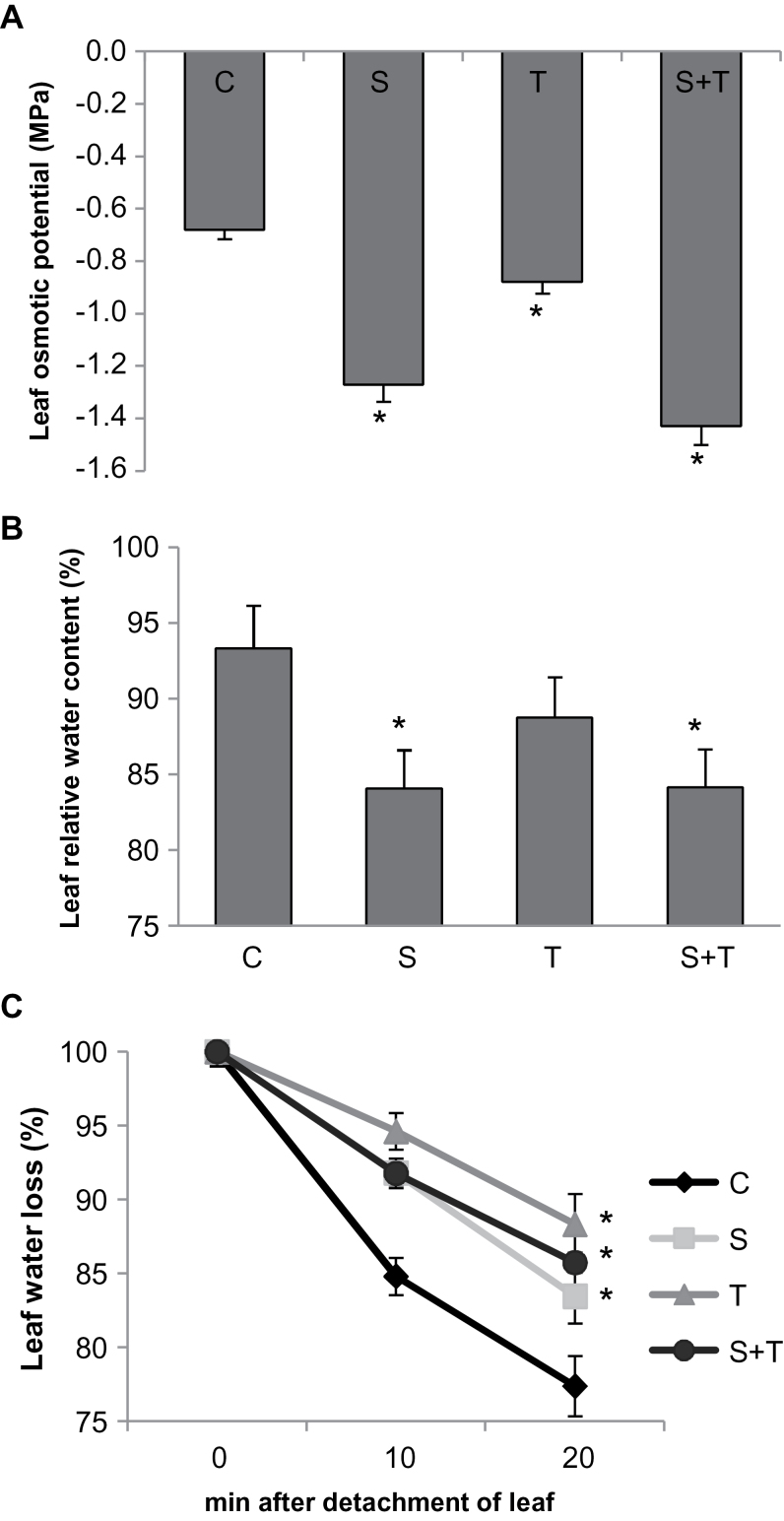
Physiological parameters in controls and plants treated with salinity and/or tunicamycin. (A) Leaf osmotic potential. (B) Leaf relative water content. (C) Leaf water loss. *Significant difference from control (*P*<0.05). C, control; S, salinity; T, tunicamycin.

The highest decrease in osmotic potential was observed under the combination of salt and Tm, whereas Tm alone caused a 28% decrease in osmotic potential (Fig. 2A). The relative water content of salt-treated plants decreased by 9%, while Tm alone reduced relative water content by only 4% (Fig. 2B).

Under adverse environmental conditions, plants close their stomata to inhibit rapid water loss (Ozfidan *et al.*, 2013). To see if Tm has an effect on stomatal closure, leaf water loss was determined. As seen in Fig. 2C, leaf water loss of control plants was the highest, while all three stress treatments (Tm, salt, and Tm and salt) decreased water loss from the leaves.

### Salt stress and Tm induced the expression of ER-stress-related genes and ER-membrane-localized *ERO1*

**Fig. 3.**
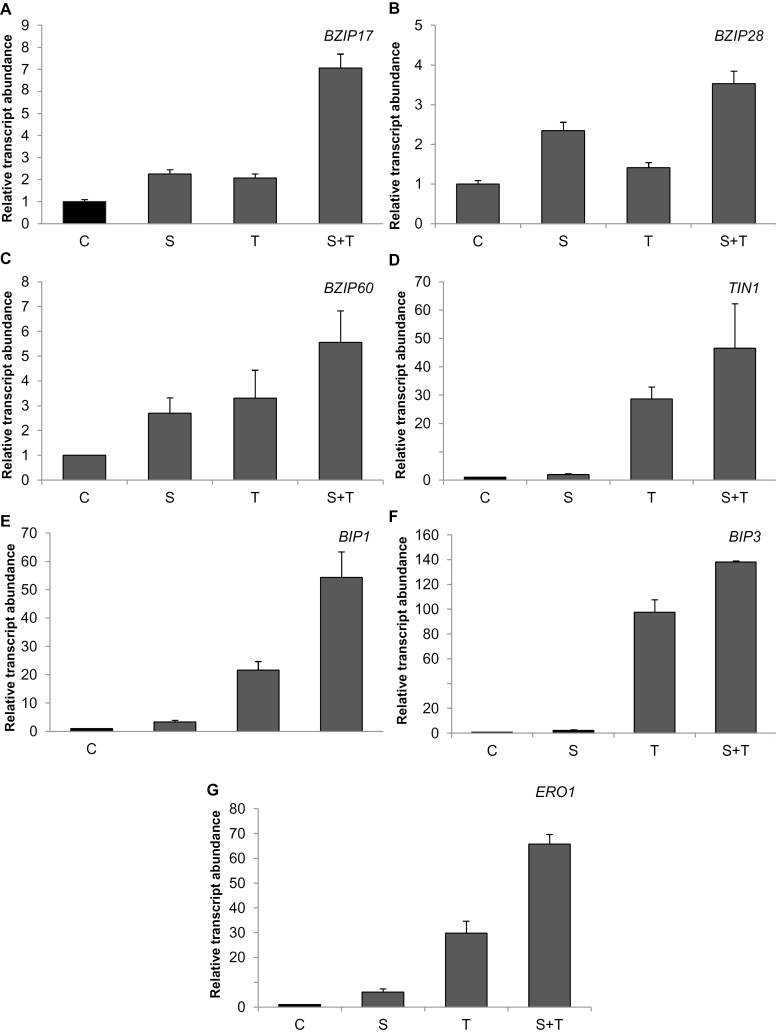
qRT-PCR results for expression of *BZIP17*, *BZIP28*, *BZIP60*, *BIP1*, *BIP3*, *TIN1*, and *ERO1* in shoots of controls and plants treated with salinity and/or tunicamycin. Expression was normalized using *Actin8*. Control plants were used as a reference point (set to 1). C, control; S, salinity; T, tunicamycin.

To understand if ER-stress-related genes were affected from oxidative damage caused by salt stress, expression of *bZIP17*, *bZIP28*, *bZIP60*, *TIN1*, *BiP1*, *BiP3*, and *ERO1* were detected under salt, Tm, and the combination of these two stresses (Fig. 3). *bZIP17* and *bZIP28* are responsible for coding of transcription factors which are proteolytically activated by ER stress (Tajima *et al.*, 2008; Deng *et al.*, 2013). In this study, both genes were upregulated by ER stress, and the combination of salt and Tm even caused further induction of these transcripts. Among the genes investigated, the expression of *bZIP60*, which activates the transcription of ER-stress-related genes (Iwata and Koizumi, 2005), showed an increase under all treatments, and the combination of salt and Tm resulted in the most abundant expression of this gene, implying that salinity increased the severity of ER stress (Fig. 3C). Transcript abundance of ER stress markers such as *TIN1*, *BiP1*, and *BiP3* were also enhanced by Tm and the combination of salt and Tm (Fig. 3D–F). The combination of Tm and salt increased the expression of these genes more than Tm-only treatment, similarly to its effect on *bZIP60*. However, salinity-only treatment had a slight effect on the expression of these genes.

ERO1 is located in the ER membrane and catalyses disulphide bond formation, producing one molecule H_2_O_2_ per one disulphide bond (Onda *et al.*, 2009). Salinity-only treatment increased *ERO1* expression by 6-fold as compared to its control, which indicated a direct relation between salinity and ER-related H_2_O_2_ production (Fig. 3G). Similarly to the other ER-stress-related genes, the highest expression of *ERO1* was detected under the combination of salt and Tm.

### Tm induced *RBOHD* and *RBOHF*, which caused an early increase in H_2_O_2_ levels and triggered ROS signalling; the combination of salt and Tm accelerated this event

NADPH oxidase enzyme (NOXs), which is known as respiratory burst oxidases or RBOHs, is one of the enzymic complexes which is responsible for generation of apoplastic ROS (Suzuki *et al.*, 2011). Several studies have revealed that plant NADPH oxidase/RBOH takes role in a multitude of different plant processes such as lignification (Denness *et al.*, 2011), pollen tube growth (Potocky *et al.*, 2007), root hair formation (Foreman *et al.*, 2003), stomatal closure (Kwak *et al.*, 2003; Zhang *et al.*, 2008), and biotic interactions (Torres *et al.*, 2005). Moreover, RBOHs also regulate signalling in response to abiotic stresses such as heat, drought, cold, high light intensity, salinity, or wounding (Kwak *et al.*, 2003; Miller *et al.*, 2009).

**Fig. 4.**
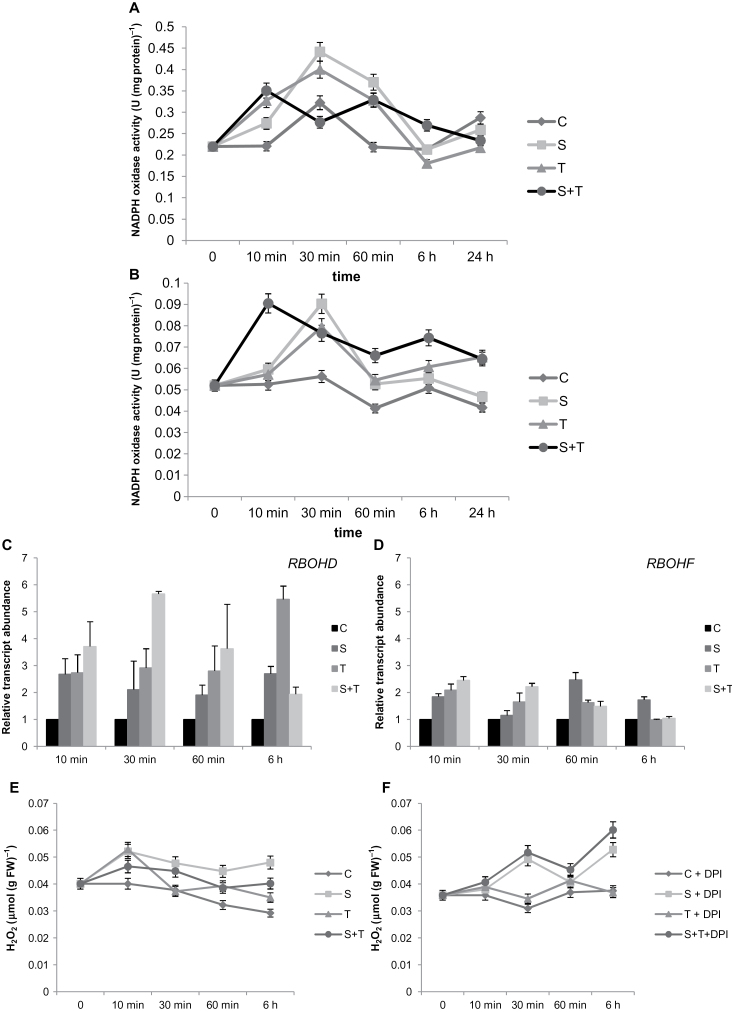
ROS-signalling-related parameters in controls and plants treated with salinity and/or tunicamycin. (A and B) NADPH oxidase activity in shoot tissue (A) and roots (B). (C and D) qRT-PCR results for *RBOHD* (C) and *RBOHF* (D) in shoots. (E and F) H_2_O_2_ content of roots without (E) or with (F) NADPH oxidase inhibitor (10 μM diphenyl iodonium, DPI). Plants were harvested at 0, 10, 30, 60min and 6 and 24h. Expression was normalized using *Actin8*. Control plants were used as a reference point (set to 1). For DPI treatment, plants were soaked in growth solution containing DPI 1h before the stress treatment. C, control; S, salinity; T, tunicamycin.

To reveal the effects of ER stress on NADPH oxidase/RBOH-mediated ROS signalling, the present study conducted time-course measurements (10, 30, 60min, and 6 and 24h) of NOX activity (Fig. 4). Salt and Tm treatments caused an induction of NOX activity at 30min in both roots and shoots (Fig. 4A, B). However, when these two stresses were combined, this induction was observed at 10min in roots (Fig. 4B). The combination of Tm and salt caused a NOX-dependent increase in ROS accumulation 20min earlier than these two stresses alone.

*RBOHD* and *RBOHF* are known to be two of the *RBOH* genes most responsive to environmental stimuli (Suzuki *et al.*, 2011). Therefore, to better understand the NADPH oxidase/RBOH induction under ER stress, the present study investigated the expression levels of these two genes. All treatments increased the expression of *RBOHD* and *RBOHF* (Fig. 4C, D). The highest expression of *RBOHD* was observed at 30min of Tm with NaCl treatment and at 6h of Tm treatment. *RBOHD* expression was more responsive to Tm-induced ER stress and the combination of Tm with NaCl as compared to *RBOHF*.

In order to see the extent of H_2_O_2_ production by NADPH oxidase/RBOH, diphenyl iodonium (DPI) inhibition studies were conducted on roots. In the first 10min, an increase in accumulation of H_2_O_2_ was observed (Fig. 4E), and this increase was inhibited by DPI (Fig. 4F). These results proved that the accumulation of H_2_O_2_ in response to ER stress was caused by NADPH oxidase/RBOH.

### ER stress induced the antioxidant system

To investigate whether ER stress causes an induction on antioxidant system of *A. thaliana*, the activities of key antioxidant enzymes, such as SOD, CAT, APX, and GR, and nonenzymic antioxidants, such as GSH, were measured in leaves and roots under both ER stress, salinity, and their combination.

**Fig. 5.**
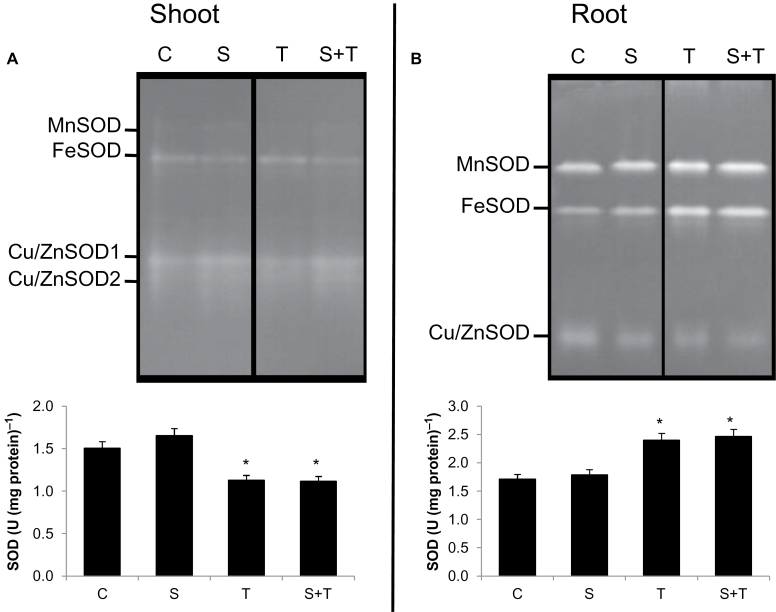
Superoxide dismutase (SOD) isoenzyme patterns and total activities in controls and plants treated with salinity and/or tunicamycin. (A) Shoot tissue. (B) Roots. *Significant difference from C at *P*<0.05. C, control; S, salinity; T, tunicamycin.

Salinity enhanced SOD activity of shoots by 10% as compared to controls while Tm treatment decreased its activity by 25%. The combination of salt and Tm decreased SOD activity by 26% as compared to controls (Fig. 5A). Salt treatment did not affect SOD activity of roots whereas Tm treatment enhanced it by 40%, and the combination of salt and Tm increased SOD activity by 43% (Fig. 5B).

Four SOD isoenzymes (MnSOD, FeSOD, and two Cu/ZnSOD) were identified in shoots. Among of these isoenzymes, FeSOD activity was the one most reduced by the combination of salt and Tm. On the other hand, in roots, three SOD isoenzymes were identified (MnSOD, FeSOD, Cu/Zn SOD), and MnSOD and FeSOD were highly enhanced by Tm.

**Fig. 6.**
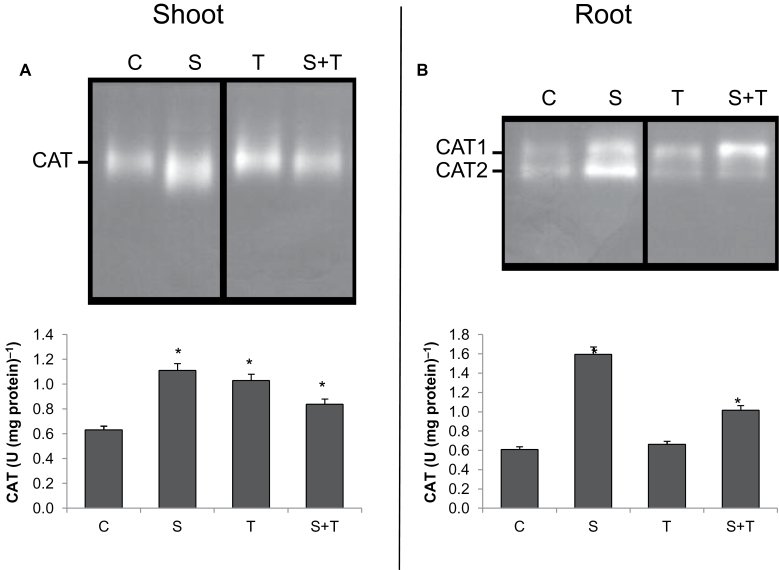
Catalase (CAT) isoenzyme patterns and total activities in controls and plants treated with salinity and/or tunicamycin. (A) Shoot tissue. (B) Roots. *Significant difference from C at *P*<0.05. C, control; S, salinity; T, tunicamycin.

In shoots, all treatments increased CAT activity as compared to controls. A higher induced activity of CAT under stress might be attributed to increased H_2_O_2_ levels. Salinity enhanced CAT activity by 76%, whereas Tm alone enhanced it by 63%, and the combination of salt and Tm increased it by 32% (Fig. 6A). However, in roots, Tm did not result in any increase on CAT activity, while salt enhanced its activity by 1.6-fold as compared to control, and the combination of Tm and salt induced CAT activity by 68% as compared to control (Fig. 6B).

Only one band for CAT, which contained a mix of different isoenzymes, was determined in shoots while two different CAT isoenzymes (CAT1 and CAT2) were found in roots. The highest CAT activity in roots was observed in salt-treated groups due to higher CAT2 activity. However, Tm decreased CAT2 activity while it did not affect CAT1 activity in roots.

### ER stress induced the Asada–Halliwell pathway in shoots and roots and enhanced GSH accumulation

**Fig. 7.**
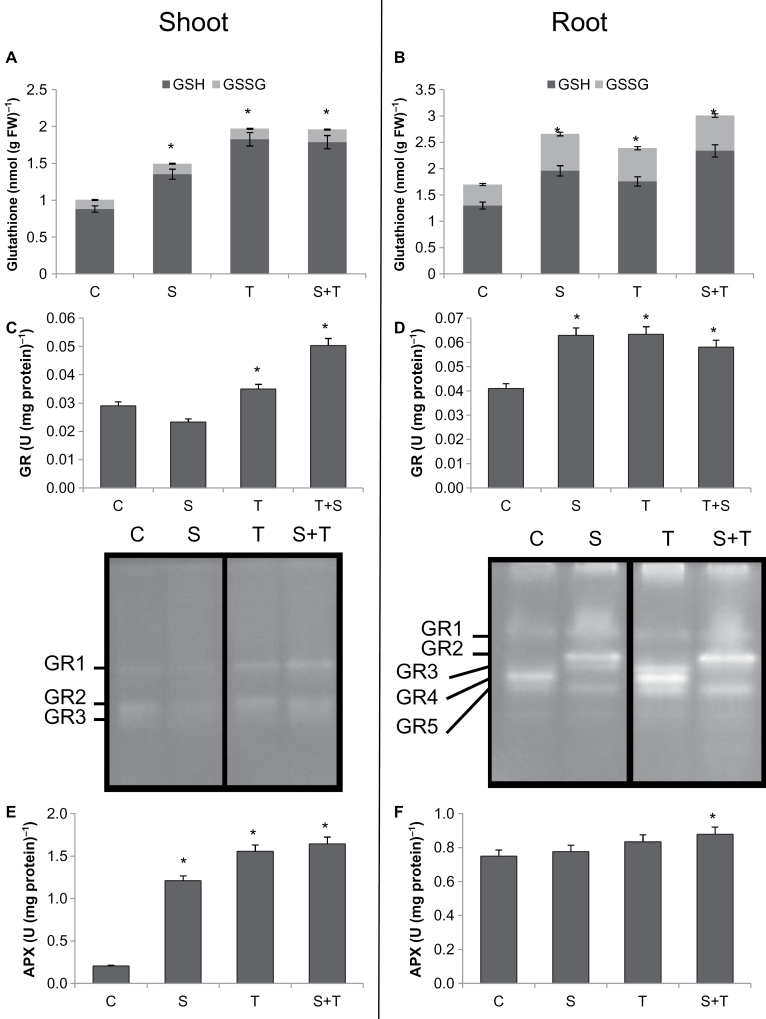
Asada–Halliwell pathway related parameters in controls and plants treated with salinity and/or tunicamycin. (A and B) GSH and GSSG contents in shoots and roots. (C and D) Glutathione reductase (GR) activity in shoots (C) and roots (D). (E and F) Ascorbate peroxidase (APX) activity in shoots (E) and roots (F). *Significant difference from C at *P*<0.05. C, control; S, salinity; T, tunicamycin.

Accumulation of unfolded proteins in ER caused increased expression of *ERO1* (Fig. 3G). hence increasing H_2_O_2_ production; as a result of this, increased ERO1 activity might induce the depletion of available GSH. To investigate ER-stress-related changes in the redox pool of the cell, the GSH/GSSG pool was measured under salinity, Tm, and their combination (Fig. 7A, B). As expected, salinity increased the total GSH pool in shoots. In the case of ER stress, the UPR caused by Tm also increased the total GSH pool by 2.2-fold in shoots and 1.35-fold in roots as compared to controls, which is, as far as is known, the first report indicating a direct link between ER stress and the redox state of the cell.

GR activity was decreased by salinity treatment, while Tm alone increased it by 17% and the combination of salt and Tm increased it by 72% in shoots, as compared to control (Fig. 7C). However, in roots, all of these treatments increased GR activity: both 80mM NaCl and Tm alone increased it by 51%, the combination of NaCl and Tm enhanced GR activity by 41% as compared to control (Fig. 7D).

Three isoenzymes of GR were identified in shoots, and activity of GR1 was enhanced by Tm treatment. Five isoenzymes were detected in roots (Fig. 7D). With salt, GR2 activity was detected and GR4 activity was suppressed. ER stress doubled GR4 activity as compared to control.

APX activity of shoots was enhanced by all three treatments. Salinity increased it by 6-fold, whereas 7.8-fold enhancement were found in Tm-treated shoots (Fig. 7E). Tm and salinity together increased APX activity by 8.2-fold as compared to control. Stress treatments also increased APX activity in roots (Fig. 7F).

### Tm-induced ER stress caused oxidative damage, as evident by increased accumulation of H_2_O_2_, lipid peroxidation, and protein oxidation

**Fig. 8.**
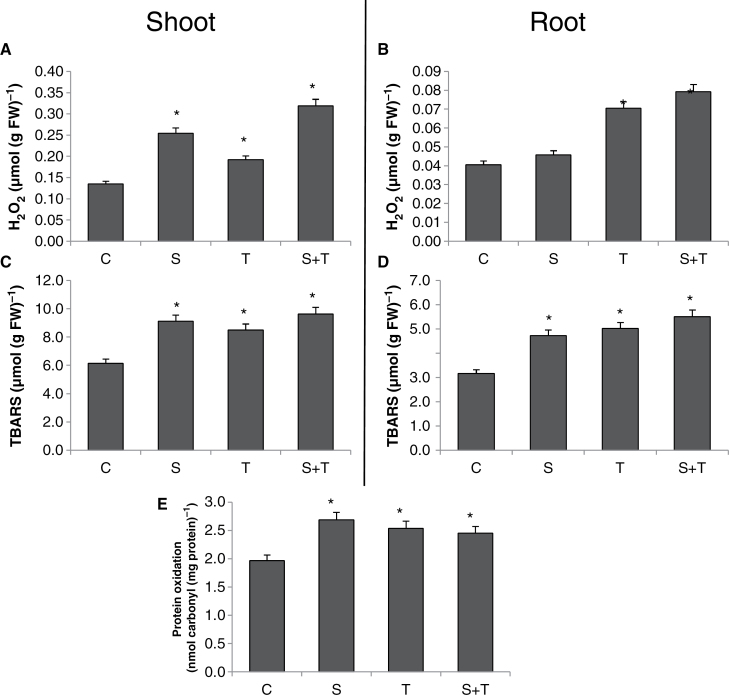
Parameters related to ROS content and oxidative damage in controls and plants treated with salinity and/or tunicamycin. (A and B) H_2_O_2_ content of shoots (A) and roots (B). (C and D) Lipid peroxidation levels in shoots (C) and roots (D). (E) Protein oxidation level in shoots. *Significant difference from C at *P*<0.05. C, control; S, salinity; T, tunicamycin.

H_2_O_2_ is an important signal molecule that provokes the antioxidant defence system to deal with ROS-mediated cell damage. However, excess amount of H_2_O_2_ can damage biomolecules (Mittler, 2002). Tm-induced ER stress caused 46% induction on formation of H_2_O_2_, whereas salt-only treatment induced its content by 92% as compared to controls in shoots. The combination of these two stresses caused a higher increase in H_2_O_2_ content of shoots and also roots (Fig. 8A and B). The difference in H_2_O_2_ levels between salinity and the combination of Tm and salt might indicate that H_2_O_2_ formation is caused by ERO1 activity. However, it is important to note that assay used in this study in part also detect lipid peroxides in addition to H_2_O_2_. Therefore these results should be evaluated with caution, but it is unlikely that the level of lipid peroxides can reach to significant level that could interfere with the results for H_2_O_2_ under such a short time span (10, 30, 60min).

Lipid peroxidation is an indicator of oxidative cell damage (Mittler, 2002). In shoots, salinity increased the TBARS content by 48%, while Tm increased it by 38% as compared to controls and the combination of salinity and Tm enhanced TBARS content by 56% as compared to control (Fig. 8C). In the case of roots, all stress treatments enhanced TBARS content, similarly to shoots: salt increased it by 49%, while Tm enhanced it by 58% and the combination of salt and Tm lead to a 74% increase in TBARS content (Fig. 8D).

Tm directly inhibits proper protein folding, but the ratio of protein oxidation caused by Tm treatment has not been determined before. Salinity, Tm, and the combination of Tm and salt increased protein oxidation by 36, 29, and 25%, respectively, in shoots (Fig. 8E).

## Discussion

The main aim of this study was to reveal the relationship between ER-stress-related oxidative stress and antioxidant defence responses in the model plant *A. thaliana* and to reveal the role of ER-originated ROS involved in stress signalling.

The ER is the main secretory protein-folding centre in the cell (Urade, 2007, 2009). Accumulation of unfolded and/or misfolded proteins are enhanced by massive protein load or environmental conditions in the ER and cause ER stress, which triggers the UPR in the cell, which can be characterized by a set of specific ER-stress genes (Liu *et al.*, 2007; Iwata and Koizumi, 2012). ER protein-folding machinery requires the formation of disulphide bonds, which causes H_2_O_2_ production, catalysed by ERO1 (Sevier and Kaiser, 2008). H_2_O_2_ has two different effects in the cell: (i) it is one of the known secondary messengers to regulate the antioxidant defence, which responds to stress conditions (Gechev *et al.*, 2002); and (ii) at some point, excess amounts of H_2_O_2_ in the cell cause irreversible damage and leads to cell death (Gechev *et al.*, 2006). Apart from these effects, the specific role of ER-originated H_2_O_2_ is not known (Jaspers and Kangasjärvi, 2010). The present study revealed that ER stress caused by Tm induces enzymic and nonenzymic antioxidants and that ER-originated H_2_O_2_ might induce the well-known antioxidant defence system to cope with stress conditions and induce an increase in ROS production to trigger ROS signalling.

To reveal the relationship between the root architecture and ER stress, this study measured main root length and lateral root density. The findings indicate a clear link between ER stress and root development. In the present study, Tm treatment showed a remarkable effect on main root length (Fig. 1). Similarly, Watanabe and Lam (2008), who determined impact of Tm-induced ER stress on *Arabidopsis* seedlings, also detected severe root growth reduction in *Arabidopsis* under ER stress due to Tm-induced classic nuclear changes in root cells that are hallmarks of programmed cell death. Moreover, earlier studies indicated that not only induced nuclear change but also glutathione depletion, which blocks auxin transport, impairs the growth of the main root. On the other hand, Reinhardt *et al.* (2003) showed that the patterning of lateral root formation is directly related to GSH and auxin patterning and/or distribution. In the present study, it was found that 0.25 and 1 μg ml^–1^ Tm-treated plants had lower lateral root densities, as compared to control. However, contrary to expectations, lateral root densities of 0.1 μg ml^–1^ Tm-treated plants were increased. This result might indicate that inhibition of GSH synthesis might not impair lateral root formation. However, there is no evidence that glutathione or auxin homeostasis is changed in Tm-treated roots; only Irsigler *et al.* (2007) found that ER stress repressed a putative auxin-amidohydrolase precursor, which might indicate a relation between auxin and ER stress.

This study observed less leaf water loss with Tm treatment. Similarly, Zhang *et al.* (2008) used a *lew1* mutant (impaired in protein folding due to inefficient glycosylation) and found that stomatal conductance and leaf water loss was lower in *lew1* as compared to wild type. Hence, these results indicate also a role for ER-originated H_2_O_2_ in stomatal closure.

*bZIP17* and *bZIP28* are genes encoding transcription factors which mediate the ER stress response in *Arabidopsis* (Tajima *et al.*, 2008). Moreover, *bZIP60* is responsible to regulate the expression of ER-stress-related genes such as *BiP1* and *BiP3* (Iwata and Koizumi, 2005, Howell, 2013, Srivastava *et al.*, 2013), and the expression of *TIN1* is distinctive to Tm treatment and an indicator of ER stress (Iwata *et al.*, 2010). In this study, to show the occurrence of the ER stress caused by Tm and the activation of UPR at the transcript level, expression of *bZIP60*, *BIP1*, *BIP3*, and *TIN1* was measured. It is known that salt stress causes H_2_O_2_ formation in the cell in several compartments such as chloroplasts and mitochondria (Miller *et al.*, 2010). However, there is no information on salinity causing H_2_O_2_ formation in the ER. The present results show that, under salinity, the ER-stress-responsive *ERO1* gene can also be induced and may contribute to H_2_O_2_ formation. This was also supported by increase in H_2_O_2_ level under Tm-only treatment.

ROS signalling can regulate calcium, protein phosphorylation, and lipid signalling (Suzuki *et al.*, 2011). NOX, the main ROS producer during oxidative signalling, have essential roles in signal transduction and perception in *Arabidopsis* (Suzuki *et al.*, 2011; Wrzaczek *et al.*, 2013). In the present study, salt and ER stress alone increased NOX activity after 30min exposition. However, under the combination of salt and Tm, this induction in NOX activity occurred 20min before (i.e. just 10min after treatment). In *A. thaliana*, 10 NOX genes have been identified, of which three (respiratory burst oxidase homologues *AtRBOHD*–*AtRBOHF*) are expressed (Dwyer *et al.*, 1996; Keller *et al.*, 1998). It was found that each RBOH isoform has very specific functions due to differences in their expression profiles in organs, tissues, and plant developmental stages such as stamen, pollen, and the root endodermis (Suzuki *et al.*, 2011). Studies have shown that ROS generated by the *A. thaliana RBOHC* regulate root hair growth through the activation of Ca^2+^ channels (Foreman *et al.*, 2003), and *RBOHD* and *RBOHF* regulate stomatal closure, seed germination, and root elongation through abscisic acid signalling (Kwak *et al.*, 2003). Genetic analysis demonstrated that reduction or lack of *RBOHD* and *RBOHF* leads to elimination of extracellular H_2_O_2_ (Torres *et al.*, 2005). Recently, Xie *et al.* (2011 and Lin *et al.* (2009) reported that salt stress triggers changes in ROS production and, in *RBOHD*, transcription of *A. thaliana* and *Zea mays*. Similarly, also in this study, *RBOHD* and *RBOHF* were induced under salt stress. However, the present study, for the first time as far as is known, shows that Tm alone can also induce expression of these genes. Moreover, the combination of salinity and Tm showed a synergistic effect on expression of *RBOHD*. According to this, early induction of NOX activity might be attributed to higher expression of *RBOHD* and *RBOHF* due to the combined stress (Fig. 4). These findings suggest that the NOX-mediated ROS accumulation caused by Tm that can be inhibited by DPI (Fig. 4F) might play a role in induction of UPR and other defensive responses such as antioxidant system. Moreover, NOX activity in the guard cells mediates stomatal closure (Kwak *et al.*, 2003). Hence, lower water losses in Tm-treated plants without any additional osmoticum in the growth medium can be explained with this ROS signalling caused by increased NOX activity.

It is known that environmental stresses such as salinity and drought induce antioxidant defence mechanisms (Turkan and Demiral, 2009). SOD, APX, and CAT are the main ROS scavenging enzymes that keep the cell in oxidative balance (Mittler, 2002). SOD, one of the well-known antioxidant defence components of the cell, catalyses the conversion of ${\text{O}}_{\text{2}}^{.-}$ to H_2_O_2_, and CAT is the main scavenger of H_2_O_2_ (Mittler *et al.*, 2011). In this study, the SOD activity in roots increased by ER stress might be related to an ER-stress-associated oxidative imbalance of the cell. However, a boost in SOD activity was not observed in shoots, even though an increase in CAT activity was observed with the ER-stress inducer Tm. A higher requirement of ATP for increased proteolytic degradation of mis/unfolded proteins might result in disturbances in electron transport of mitochondria, causing increased ${\text{O}}_{\text{2}}^{.-}$ production. This series of events can explain the increase in MnSOD activity, which is located in mitochondria, in roots of Tm-treated plants.

Due to accumulation of unfolded proteins in ER, UPR was activated (Fig. 3) and expression of *ERO1* was increased (Fig. 3G) in Tm-treated plants. To reduce or rearrange incorrect disulphide bonds in proteins, GSH is used in the ER lumen (Tu and Wiesman, 2004). To prevent GSH depletion in the ER, GR activity increased in Tm-treated plants and also the size of the total GSH pool was enhanced. Additionally, a change in GR isoenzyme pattern was observed with Tm treatment, suggesting expression of a new set of GR isoforms due to ER stress. APX is one of the important H_2_O_2_ scavengers in the cell, especially in the chloroplast and cytoplasm (Asada, 2006). Environmental stress conditions enhance APX activity due to stress-related ROS production (Miller *et al.*, 2010). In shoots, APX showed the highest increase than all other measured antioxidant enzymes, and Tm-related ER stress made a significant impact on APX activity. Regeneration of the substrate of APX, ascorbate, is ultimately linked to GSH pool via dehydroascorbate reductases. Therefore, induction of GSH synthesis and GR enzyme supports the ROS scavenging capacity of APX during salinity and ER stress.

H_2_O_2_ accumulation by stress causes ROS-induced lipid peroxidation, which is widely used as an oxidative stress marker (Mittler, 2002). Data presented in the study suggest that Tm treatment can cause oxidative damage on lipids. In addition to lipid peroxidation, protein carbonyl groups are also used as an oxidative marker (Levine *et al.*, 1994). Accumulation of unfolded proteins in ER can make polypeptides more susceptible to oxidative modifications due to exposure of interior portions of unfolded proteins. This hypothesis was supported by the fact that Tm treatment increased the protein oxidation levels in the cell.

The results from this study imply that Tm-induced H_2_O_2_ production can signal to induce the antioxidant defence system and balance the redox status of the cell in *Arabidopsis*. Hence, there might be an interaction between antioxidant defence system and UPR to suppress ER-originated ROS. Data about NADPH oxidase/RBOHs show that ROS signalling caused by ER stress might play a role in induction of UPR in addition to its effect on stomatal closure. These results help to understand the relationship between ER stress and oxidative stress, redox status, antioxidant defence, and ROS signalling for the first time in a plant system.

## Supplementary material

Supplementary data are available at *JXB* online.

Supplementary Table S1. Gene names and oligonucleotide sequences for quantitative RT-PCR primers used in the study.

Supplementary Data
