# Supplementary material for: Endoplasmic reticulum stress triggers ROS signalling, changes the redox state, and regulates the antioxidant defence of Arabidopsis thaliana
Source: J Exp Bot. 2014 Feb 20;65(5):1377–90. doi: 10.1093/jxb/eru034 (PMC3969530; doi:10.1093/jxb/eru034)
Supplement: Supplementary Data [file supp_65_5_1377__index.html]

Endoplasmic reticulum stress triggers ROS signalling, changes the redox state, and regulates the antioxidant defence of Arabidopsis thaliana — Endoplasmic reticulum stress triggers ROS signalling, changes the redox state, and regulates the antioxidant defence of Arabidopsis thaliana — Supplementary Data 

# Endoplasmic reticulum stress triggers ROS signalling, changes the redox state, and regulates the antioxidant defence of *Arabidopsis thaliana*

## Supplementary Data

Data files

**Files in this Data Supplement:**

- Supplementary Data - Supplementary Data
